# Cortical modulation of short-latency TMS-evoked potentials

**DOI:** 10.3389/fnhum.2012.00352

**Published:** 2013-01-09

**Authors:** Domenica Veniero, Marta Bortoletto, Carlo Miniussi

**Affiliations:** ^1^Neuroscience Section, Department of Clinical and Experimental Sciences, University of BresciaBrescia, Italy; ^2^Cognitive Neuroscience Section, IRCCS Centro San Giovanni di Dio FatebenefratelliBrescia, Italy

**Keywords:** transcranial magnetic stimulation, electroencephalography, TMS–EEG, premotor cortex, non-invasive brain stimulation, NIBS, motor-evoked potentials, motor cortex

## Abstract

Transcranial magnetic stimulation–electroencephalogram (TMS–EEG) co-registration offers the opportunity to test reactivity of brain areas across distinct conditions through TMS-evoked potentials (TEPs). Several TEPs have been described, their functional meaning being largely unknown. In particular, short-latency potentials peaking at 5 (P5) and 8 (N8) ms after the TMS pulse have been recently described, but because of their large amplitude, the problem of whether their origin is cortical or not has been opened. To gain information about these components, we employed a protocol that modulates primary motor cortex excitability (MI): low frequency stimulation of premotor area (PMC). TMS was applied simultaneously with EEG recording from 70 electrodes. Amplitude of TEPs evoked by 200 single-pulses TMS delivered over MI at 110% of resting motor threshold (rMT) was measured before and after applying 900 TMS conditioning stimuli to left PMC with 1 Hz repetition rate. Single subject analyses showed reduction in TEPs amplitude after PMC conditioning in a sample of participants and increase in TEPs amplitude in two subjects. No effects were found on corticospinal excitability as recorded by motor-evoked potentials (MEPs). Furthermore, correlation analysis showed an inverse relation between the effects of the conditioning protocol on P5-N8 complex amplitude and MEPs amplitude. Because the effects of the used protocol have been ascribed to a cortical interaction between premotor area and MI, we suggest that despite the sign of P5-N8 amplitude modulation is not consistent across participant; this modulation could indicate, at least in part, their cortical origin. We conclude that with an accurate experimental procedure early latency components can be used to evaluate the reactivity of the stimulated cortex.

## Introduction

Combining transcranial magnetic stimulation (TMS) with electroencephalogram (EEG) recording makes possible to test the reactivity of brain areas, i.e., the cortical response to the magnetic pulse (Ilmoniemi et al., 1997), by means of TMS-evoked potentials (TEPs), which are directly generated by the cortex and provide a marker of the brain state (for a review, see Komssi and Kähkönen, 2006; Miniussi and Thut, 2010).

Despite their reproducibility across different studies (Komssi et al., 2002, 2004; Kähkönen et al., 2004; Bonato et al., 2006; Lioumis et al., 2009; Ferreri et al., 2011; Busan et al., 2012), TEP components are still not completely understood, and both their functional meaning and cortical origin are highly debated. Moreover, EEG analyses have often been restricted to several ms after the TMS pulse, i.e., starting from 10 ms (Komssi et al., 2002; Litvak et al., 2007), or even longer intervals. Recently, short-latency TEPs peaking at 5 and 8 ms after TMS pulse (P5 and N8, respectively) have been described (Bonato et al., 2006; Esser et al., 2006; Veniero et al., 2010; Ferreri et al., 2011). In particular, Veniero et al. (2010) showed that when TMS is applied over primary motor cortex (MI), P5, and N8 components reach their peak over motor areas and can be modulated by the frequency of stimulation, suggesting that they might represent the direct response of the stimulated motor cortex. Because P5 and N8 are large signal deflections, their cortical origin has remained uncertain. However, data suggest that P5 and N8 are not residual magnetic artifacts and that they cannot be fully explained by spurious muscle activation (Veniero et al., 2010; but see Mäki and Ilmoniemi, 2011). To gain further information about these components, we designed a protocol to modulate MI excitability by means of premotor cortex (PMC) stimulation. The effects induced by this type of protocol have been ascribed to a cortical phenomenon, i.e., a change in excitability of the circuits within MI after PMC stimulation (Gerschlager et al., 2001; Munchau et al., 2002; Rizzo et al., 2004; Suppa et al., 2008). Therefore, a modulation of P5 and N8 components would support their cortical origin.

## Materials and methods

### Participants

Fifteen right-handed healthy volunteers participated in the study. Two participants were excluded from the final analysis due to excessive noise in the EEG recording. The remaining 13 participants (8 males and 5 females) aged between 18 and 30 years. None had a history of psychiatric, neurological or other relevant medical disease or any contraindication for TMS (Rossi et al., 2009). The protocol was performed in accordance with ethical standards and approved by the CEIOC Ethics Committee of IRCCS Centro San Giovanni di Dio Fatebenefratelli, Brescia, Italy. Informed consent was obtained from participants prior to the beginning of the experiment.

### Procedure

Participants were comfortably seated on an armchair with the right arm in a resting position, looking at a fixation cross in front of them. TMS was delivered using a Super Rapid transcranial magnetic stimulator connected to four booster modules and a double 50-mm figure-eight custom coil (Magstim Company, Whitland, UK). The coil was placed tangentially to the scalp, with the longer axes perpendicular to the central sulcus. The hot-spot was defined as the point at which the TMS induced the maximum motor-evoked potentials (MEPs) from the relaxed right first dorsal interosseous (FDI), and the resting motor threshold (rMT) was defined as the TMS intensity eliciting MEPs of at least 50 μV in 5 out of 10 trials (Rossini et al., 1994).

**Figure 1:**
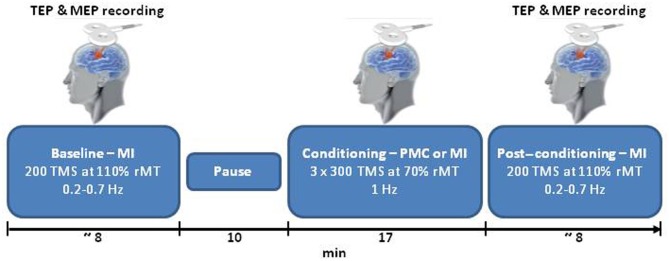
**A schematic representation of the experimental procedure.** TEPs and MEPs evoked by single pulse TMS over MI were collected for each participant before (baseline) and after (post-conditioning) TMS at 1 Hz (conditioning). In the main condition TMS was applied over PMC; MI was chosen as control site. PMC and MI conditioning were performed in two separate days. TMS, transcranial magnetic stimulation; MEP, motor-evoked potentials; MI, primary motor cortex; PMC, premotor cortex; TEP, TMS-evoked potentials; and the rMT, resting motor threshold.

Each session started with TMS over the left MI (pre-conditioning block), followed by a 10-min rest period as displayed in Figure 1. The conditioning stimulation was then applied. Finally, TMS was delivered over the left MI (post-conditioning block). During the pre- and post-conditioning blocks, 200 single TMS pulses were delivered at random intervals (0.2–0.7 Hz) at 110% of the rMT, which ensured a high signal to noise ratio. During the conditioning stimulation, 1 Hz TMS was delivered in three blocks of 300 stimuli at 70% of the rMT, interspersed with 1-min periods of no TMS (for a total of 900 stimuli). Two sites were conditioned: the PMC and the MI (as a control site). For PMC stimulation, the coil was moved 8% of the nasion-inion distance anteriorly from the MI hot-spot (Munchau et al., 2002). When stimulating MI the closest electrodes to the hot-spot were C3 and C1 in all subjects. During the conditioning block over PMC, 10 subjects had the stimulated area close to FC3 and FC1, and three subjects had the spot over a site located among FC3-FC1-F3. The position of the coil was controlled with a TMS neuronavigation system (SofTaxic, E.M.S., Bologna, Italy) via a graphic user interface and a 3D optical digitiser (NDI, Polaris Vicra, Ontario, Canada) to keep a high degree of reproducibility and accuracy throughout the experimental sessions (Cincotta et al., 2010; Carducci and Brusco, 2012). Two sessions at least 1 week apart were run for PMC and MI conditioning in counterbalanced order across participants. To reduce auditory contamination of EEG induced by coil clicks, subjects wore earplugs during the entire experiment.

### TEP and MEP recordings

During the pre- and post-conditioning blocks, EEG, electrooculogram (EOG), and electromyogram (EMG) were acquired (BrainAmp, Brain Products GmbH, Munich, Germany). EEG was recorded from 70 scalp electrodes using electrodes mounted on an elastic cap following the International 10-10 system of EEG sensor placement. The ground electrode was positioned in Fpz, while referenced to TP10. Horizontal and vertical eye movements were detected by EOG. The voltage between two electrodes located to the left and right of the external canthi recorded horizontal eye movements. The voltage difference between reference electrodes and electrodes located beneath the right eye recorded vertical eye movements and blinks. MEPs were recorded from the right FDI via surface electrodes in the belly tendon-montage. Skin/electrode impedance was maintained below 5 kΩ. Data were digitized at 5000 Hz and bandpass filtered between 0.01 and 1000 Hz (for recording details see Veniero et al., 2009).

**Figure 2:**
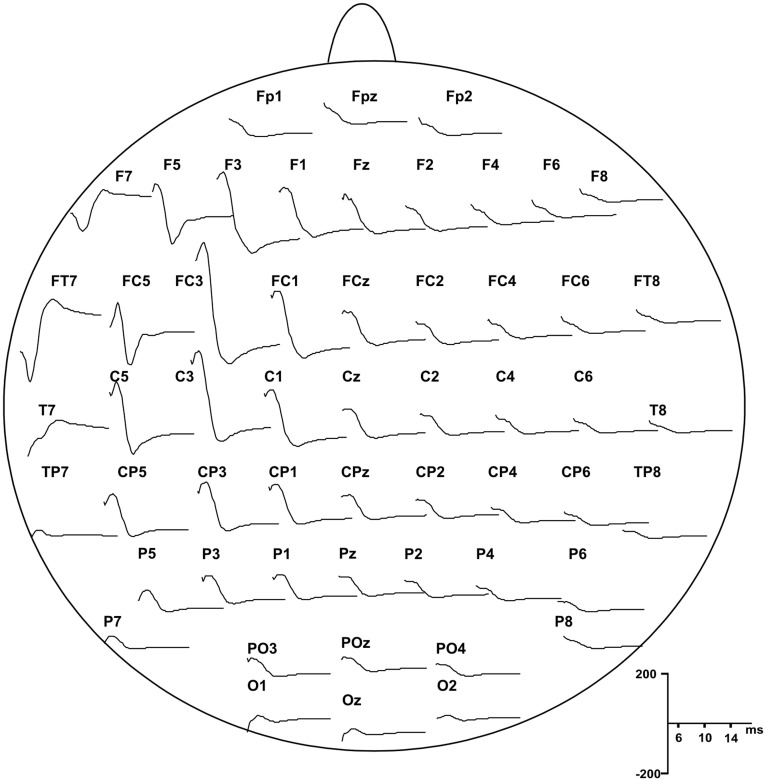
**Grand average of the TEPs responses recorded from all subjects showing scalp distribution of P5 and N8 components, starting from 5 ms after the pulse delivery**.

EEG was re-referenced offline to the average signal of TP10 and TP9. For the analysis of cortical and peripheral responses to TMS, the continuous EEG, EOG, and EMG signals were divided off-line into epochs from 100 ms before the TMS pulse (baseline) to 500 ms after, and were baseline corrected. Before averaging, all epochs were visually inspected to exclude excessively noisy EEG, eye-movement artifacts in the EOG or muscle artifacts in the EEG and EMG. To obtain the cortical evoked responses to TMS (i.e., TEPs) the epochs were averaged for each subject and condition. P5 and N8 showed the same topography with opposite polarity (see Figure 2) and were similarly modulated by our protocol; therefore we considered that they may represent a unique TEP complex, i.e., P5-N8 complex.

### Data analysis

P5 and N8 components were calculated as the average signal of five electrodes (F3, FC5, FC3, FC1, and C3) and defined as positive peak between 5 and 7 ms and negative peak between 7 and 10 ms, respectively. MEPs were measured on the same trials as peak-to-peak amplitude in the EMG signal.

To test for cortical modulation of short-latency TEPs induced by PMC or MI conditioning, a 2 by 2 repeated-measures ANOVA was performed, considering the peak to peak amplitude of P5-N8 complex. We tested for significant effects for the factors Conditioning (MI and PMC) and Time (pre-and post-conditioning). The same analysis was run to test for MEP modulation. Additionally, we performed a factorial ANOVA on each subject's data to test for significant modulations of P5-N8. The normal distribution of P5-N8 amplitude was tested using the Kolmogorov–Smirnov test (for all *p* > 0.20). When appropriate, the Greenhouse-Geisser correction was used, and *post-hoc* comparisons were Bonferroni corrected. We verified that the intensity of stimulation did not change between sessions through a paired *t*-test.

Moreover, we investigated whether PMC conditioning effects on the P5-N8 complex correlated with the effects on the MEP amplitude. We calculated the difference in P5-N8 amplitude between pre- and post-PMC conditioning and divided the result by the mean P5-N8 complex amplitude in the pre- and post-conditions. We applied the same procedure to the MEPs and submitted the data to a Pearson correlation analysis. Considering the high between-subjects variability of TEPs and MEPs, we applied this procedure to ensure that the effects of the conditioning protocol were not influenced by the absolute amplitude of P5-N8 in a single participant. With this analysis we are able to investigate if cortical components and peripheral measures of cortical reactivity, i.e., MEPs, are linearly related so that the participants who show the biggest PMC conditioning effects on the TEPs are also the participants who show the biggest effects on the MEPs.

## Results

Mean TMS intensity during conditioning was 63.3 ± 7.6% in the PMC session and 63.8 ± 7.1% in the MI session [no difference between sessions: *t*_(12)_ = 1.05, *p* = 0.32].

**Figure 3:**
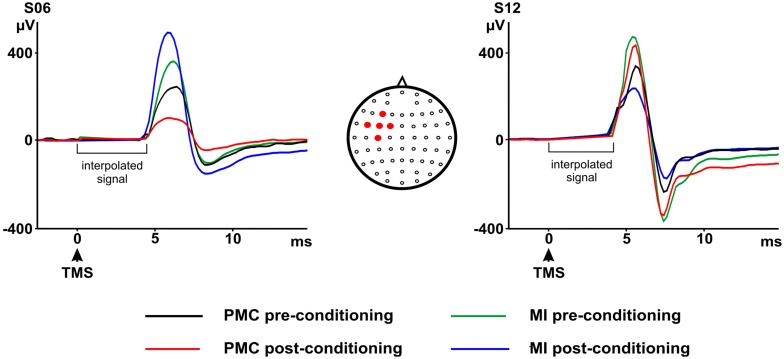
**P5 and N8 pre- and post-primary motor cortex (MI) or premotor cortex (PMC) conditioning as recorded from the marked electrodes.** On the left a representative participant showing a decrease in P5 and N8 amplitudes after PMC conditioning and an increase in P5 and N8 amplitudes after MI conditioning. On the right a representative participant showing an increase in P5 and N8 after PMC conditioning and the opposite result after MI conditioning. Electrodes montage is also shown on the upper right side of the figure. Filled circles indicate five electrodes from which the P5-N8 average signal was calculated.

The group analyses did not reveal any significant effect of the TMS conditioning paradigm on MEPs and on P5-N8 amplitude. Baseline values for MEPs and TEPs were not different across conditions [MEPs: *t*_(12)_ = 0.35, *p* > 0.05; P5-N8: *t*_(12)_ = 0.20, *p* > 0.05]. No significant main effect of Conditioning [*F*_(1, 12)_ = 0.16, *p* > 0.05] or significant Conditioning by Time interaction [*F*_(1, 12)_ < 0.01, *p* > 0.05] emerged for MEPs (pre-MI: 896.60; post-MI: 905.78; pre-PMC: 832.67 post-PMC: 841.76). P5-N8 showed a decrease in amplitude after PMC conditioning (pre-PMC: 365.98; post-PMC: 276.90) but not after MI conditioning (pre-MI: 340.21 post-MI: 347.31). However this result was not statistically significant—nor as main effect of Conditioning [*F*_(1, 12)_ = 0.06, *p* > 0.05] neither as Conditioning by Time interaction [*F*_(1, 12)_ < 0.81, *p* > 0.05], suggesting that the TMS conditioning protocol may have induced subtle or inconsistent effects across subjects. Accordingly, single subject analyses showed that the PMC conditioning was effective, by significantly modulating TEPs (P5-N8), in 8 out of 13 participants: TEPs amplitude was reduced in six participants (Figure 3) [Conditioning by Time interaction, s01: *F*_(1, 667)_ = 1887.28, *p* < 0.05; s02: *F*_(1, 702)_ = 2941.29, *p* < 0.05; s04: *F*_(1, 467)_ = 281.93, *p* < 0.05; s05: *F*_(1, 420)_ = 71.47, *p* < 0.05; s13: *F*_(1, 305)_ = 122.58, *p* < 0.05; Main effect Time: s11: *F*_(1, 493)_ = 19.81, *p* < 0.05; all *post-hoc p* < 0.05] and increased in two participants [Conditioning by Time interaction, s03: *F*_(1, 553)_ = 9.22, *p* < 0.05; s09: *F*_(1, 558)_ = 287.10, *p* < 0.05; all *post-hoc p* < 0.05] after PMC conditioning. Opposite or null results in different subjects suggest that the TMS conditioning did not have a consistent effect across subjects and may have been ineffective in some participants. Noteworthy, the P5-N8 modulation after PMC conditioning was significantly stronger than the effect of MI conditioning, as indicated by significant interactions Conditioning by Time in seven subjects, therefore suggesting that such modulations were related to the specific stimulation of PMC.

**Figure 4:**
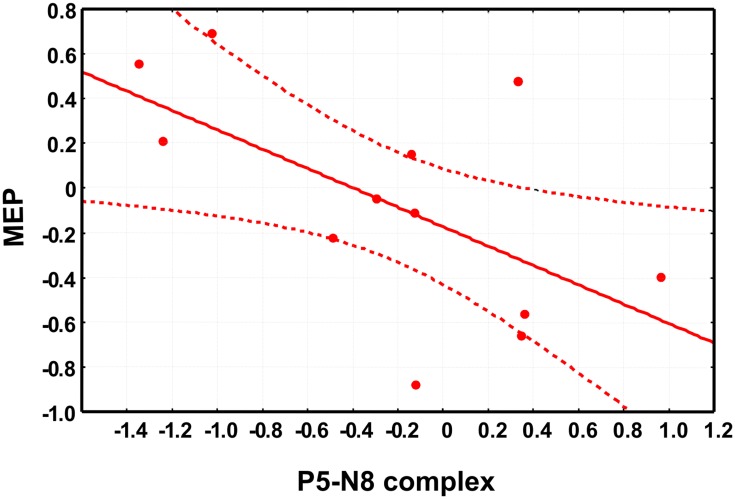
**The scatter plot shows the significant negative correlation between the changes in motor-evoked potentials (MEPs) amplitude, on the x-axis and the changes in P5-N8 complex amplitude.** Note that negative values indicate a reduced amplitude of P5-N8 complex or a reduction of MEPs amplitude after the conditioning session (see main text for details about data analysis).

Moreover, to address the question on the cortical origin of early TEPs, we took an additional approach by investigating the correlation between P5-N8 and the MEPs amplitude. Importantly, we found that the modulation of P5-N8 complex after PMC conditioning correlated with the modulation of MEPs (*r* = −0.60, *p* < 0.05) so that the stronger the decrease of P5-N8 complex amplitude, the higher the increase of MEPs. In other words, the participants showing reduced P5-N8 amplitude after PMC conditioning, showed increased MEP amplitude, and vice versa, participants showing increased P5-N8 amplitude showed decreased MEP amplitude (Figure 4). The correlation between the modulation of P5-N8 complex after MI conditioning and the modulation of MEPs was not significant (*r* = 0.41, *p* > 0.05).

## Discussion

The aim of the present study was to provide new information about two short-latency TEPs, namely P5-N8, by indirectly manipulating MI excitability. Although applying an inhibitory protocol to PMC did not consistently change MI activation across all subjects, we were able to study the relationship between P5-N8 and peripheral measures of cortical excitability with two specific analyses: single subject analyses and correlation between P5-N8 and MEPs.

Results from the single subjects analysis indicated a significant modulation of P5-N8 complex when conditioning session was performed over PMC, but not over MI. Given that both sites of stimulation are close to the facial muscles it is highly unlikely that we were stimulating facial muscles in PMC condition and not in the MI, thus we can exclude that we are simply manipulating the responses of the facial muscles. Moreover, it is unlikely that results are influenced by those artifact generated by the stimulation of the scalp, because pre- and post-conditioning session have been performed in the same way, so the artifactual activity in all session is likely to be the same. Last, additional somatosensory activation generated by the muscle twitch should be very unlikely to be involved in the generation of the early TEPs because the afferent response takes about 20 ms to reach the cortex.

By correlating the modulation of P5-N8 with the MEPs modulation, we were able to show that the P5-N8 complex shares an inverse linear relationship with the MEPs, i.e., the peripheral measure of cortical reactivity. Therefore, P5-N8 complex may represent an inhibitory process initiated by the premotor area. Indeed, our data show that the bigger was the conditioning effect on these early components, the stronger was the inhibition over MI. Accordingly, some recent TMS–EEG studies (Esser et al., 2006; Ferreri et al., 2011) localized the cortical source of both P5 and N8 in the PMC. Moreover, it has been shown that a magnetic pulse delivered over the ipsilateral PMC at 2–15 ms prior to a second stimulus over MI can reduce the MEPs amplitude, with the biggest effect at 6 ms (Civardi et al., 2001).

In contrast with previous studies, the effect of the PMC conditioning on MEP was not significant over all subjects, as indicated by no change in MEPs and TEPs amplitude in the group analyses. These results may depend on the TMS intensity (70% rMT) set during conditioning. Such a low intensity was chosen to avoid MI stimulation during the PMC conditioning and was comparable to intensities used in previous studies (Gerschlager et al., 2001; Munchau et al., 2002). Moreover, because of the PMC localization method used, it might be that the same area was not precisely targeted in all subjects. The spatial precision of the optical TMS neuronavigation system in the localization of the target area is generally a few millimetres (Herwig et al., 2001; Julkunen et al., 2008; Cincotta et al., 2010). Nevertheless this spatial precision is dependent on the resolution of the MRI data. In this case, the coil was moved 8% of the nasion-inion distance anteriorly from the MI hot-spot and it is likely that the actual TMS target site differed across subjects (Sack et al., 2009). For these reasons, the conditioning stimulation may have been ineffective in some participants, in turn leading to opposite or null results in different subjects.

The present study shows that P5 and N8 can be modulated by cortical phenomena such as the PMC conditioning at least at single subject level. Therefore, according to previous studies reporting an early TEPs modulation after paired pulse TMS (Ferreri et al., 2011) and 20 Hz rTMS (Veniero et al., 2010), it is possible to conclude that different protocols classically designed to modulate MI excitability have an impact over P5-N8 amplitude. Importantly, despite not consistently manipulated by the protocol, the amplitude of the early components was correlated to MEPs amplitude. Therefore, in our view these results points to a cortical involvement in P5 and N8 generation. However, some studies (Julkunen et al., 2008; Mutanen et al., 2012) have linked the large responses recorded after few ms from the stimulus to an exclusively artifactual phenomenon. In the present study we cannot totally exclude an involvement of muscular activity, indeed non-cortical phenomena induced by our protocol, e.g., the repeated stimulation of facial muscles, should also be considered because they can affect early TEPs. Moreover, the modulation of P5-N8 appears to have a significant inter-individual variability that should be further explored. A possible parsimonious explanation is that the P5-N8 complex may represent a cortical response together with muscular activation and that the influence of this second component on the recorded signal may vary across individuals. This would be in line with recent findings by Mutanen et al. (2012) showing that the amplitude of muscular artifact recorded during EEG-TMS experiment depends on coil rotation, tilt angles and stimulation intensities. These parameters are not constant across subjects because when MI is stimulated the final coil position is chosen with the aim to evoke a reliable MEP with the lowest intensity. Possibly more sophisticated analyses, e.g., independent component analyses (ICA) and principal component analyses (PCA), can isolate the cortical component and provide a better index of cortical activity. It has however to be noted that the muscular and the cortical activation could theoretically overlap in time and it is also possible that these different components share similar brain topography. Mäki and Ilmoniemi (2011) applied PCA to TMS–EEG data to remove muscular activation, by subtracting some components according to their frequency, amplitude and topography. This procedure however resulted in a flattening of signals covering the stimulated area. More successful approach for removing large muscle artifacts from TEPs, after stimulation of lateral areas of the scalp, have been recently applied by Korhonen et al. (2011) with the enhanced deflation method.

It has to be noted that previous studies found a correlation between late TEP components and MEPs amplitude (Mäki and Ilmoniemi, 2010; Ferreri et al., 2011). However the latency of these late components, about 30 ms, is not compatible with the generation of the descending output responsible for the targeted muscle activation. On the other hand it appears more plausible that early component could reflect those activations responsible for MEPs typically recorded after 20–25 ms from the magnetic pulse.

In conclusion, we report important results about the nature of two short-latency TEPs. Because our conditioning protocol is considered to induce cortical effects (Munchau et al., 2002), the changes in P5 and N8 amplitude and their correlation with MEPs amplitude suggest that these early TEPs have a cortical component and that they can be used to evaluate the reactivity of the stimulated cortex. Despite the possibility of a residual muscular activation, our study suggests that with careful study design, namely keeping the experimental conditions comparable and considering that a muscular activation can as well be involved, these early TEP components can be informative on the reactivity of the targeted area.

### Conflict of interest statement

The authors declare that the research was conducted in the absence of any commercial or financial relationships that could be construed as a potential conflict of interest.
